# 3D printed patient‐specific drill guide for percutaneous pedicle screw fixation in lumbosacral vertebrae in dogs: A cadaveric study and clinical case report

**DOI:** 10.1111/vsu.70115

**Published:** 2026-04-28

**Authors:** Kyuhyun Yeom, Jongpil Yoon, Youngjin Jeon, Haebeom Lee, Jaemin Jeong

**Affiliations:** ^1^ College of Veterinary Medicine Chungnam National University Daejeon Republic of Korea

## Abstract

**Objective:**

To evaluate the feasibility, accuracy, and safety of percutaneous pedicle screw fixation (PPSF) in the canine lumbosacral junction using three‐dimensional (3D)‐printed patient‐specific drill guides (PSGs).

**Study design:**

Cadaveric study and clinical case report.

**Sample population:**

A total of 13 Beagle cadavers and one 5‐year‐old German Shepherd.

**Methods:**

In each cadaver, one side underwent 3D‐PSG‐assisted PPSF by a novice surgeon and freehand PPSF on the contralateral side by an experienced surgeon. Outcomes included operative time (min), fluoroscopic counts, incision length (mm), vertebral canal breach (grade 0–2; grade 2 defined as relevant), and angular deviation (degrees). The clinical case underwent unilateral biportal endoscopy laminectomy and discectomy followed by 3D‐PSG‐assisted PPSF.

**Results:**

Operative times were comparable (PSG: 22.65 ± 2.71 min; freehand: 24.82 ± 5.00 min; *p* = .139). Fluoroscopic acquisitions were fewer with 3D‐PSG (median 5 [5–8]) than freehand (9 [7–14]; *p* = .003). At L7, canal breach occurred in 1/13 (PSG) and 6/13 (freehand; *p* = .073); relevant were 1/1 and 4/6, respectively. At S1, breach rates did not differ: 3/13 (PSG) and 2/13 (freehand; *p* = 1.000); relevant were 1/3 and 1/2, respectively. Angle deviations at L7 were smaller in the 3D‐PSG (sagittal 2.58° and 5.82°, *p* = .018; transverse 6.4° and 12.8°, *p* = .036). No significant differences at S1. In the clinical case, minor breaches occurred at L7 left (medial) and S1 right (cranial). The dog exhibited normal gait at 3 months.

**Conclusion:**

3D‐PSG‐assisted PPSF at the canine lumbosacral junction was feasible, with improved screw placement accuracy at L7 and reduced fluoroscopic exposure.

**Clinical significance:**

3D‐PSGs may standardize screw trajectory control across surgeon experience levels.

## 1 INTRODUCTION

Degenerative lumbosacral stenosis (DLSS) is a common spinal disorder in dogs, particularly in large breeds, characterized by the compression of neural structures in the lumbosacral junction.^1^ The pathology involves intervertebral disc degeneration, hypertrophy of the interarcuate ligament, sacral osteochondrosis and facet joint misalignments, leading to clinical signs such as pain, pelvic limb weakness, and neurologic deficits.^2^ Surgical intervention is often required when conservative management fails to alleviate symptoms.^1^

Various surgical techniques have been employed to treat DLSS, primarily focusing on decompression and stabilization.^3^ Decompression techniques include dorsal laminectomy and discectomy, while stabilization methods range from polymethylmethacrylate (PMMA)‐assisted pin or screw fixation or locking plates, trans‐articular facet screws and pedicle screw‐rod fixation technique.^2^, ^4^, ^5^, ^6^, ^7^ Among these, pedicle screw‐rod fixation has gained popularity due to its ability to provide rigid stabilization and flexibility in screw angulation, allowing easier alignment with connecting rods.^8^, ^9^ Despite their biomechanical advantages, stabilization procedures have traditionally been performed via open approaches, necessitating wide exposure and resulting in greater soft‐tissue trauma, longer recovery, and more postoperative complication.^10^, ^11^, ^12^

Minimally invasive surgery (MIS) techniques, such as percutaneous pedicle screw fixation (PPSF), have been increasingly adopted in human medicine.^11^, ^12^ These techniques offer several advantages, including minimized damage to the dorsal musculoligamentous complex, reduced intraoperative blood loss, less postoperative pain, and shorter hospital stays.^12^ However, percutaneous pedicle screw fixation presents technical challenges, particularly due to limited visualization.^13^ This often necessitates repeated use of fluoroscopy to identify a safe insertion point, increasing radiation exposure to both the patient and surgical team, prolonging operative time, and potentially compromising screw placement accuracy.^14^

To address these challenges, three‐dimensional (3D)‐printed patient‐specific drill guides (PSGs) have been introduced as a promising solution.^15^ Accordingly, we developed a 3D‐PSG for PPSF and evaluated its feasibility in canine lumbosacral stabilization. Given the non‐crossover design of this cadaveric study, comparisons between techniques should be interpreted as exploratory rather than definitive, consistent with approaches used in previous feasibility studies evaluating guided versus freehand screw placement.^16^ The primary objective of this study was to evaluate the feasibility of applying this technique in canine lumbosacral stabilization. Additionally, we aimed to assess its accuracy and safety in comparison with a conventional freehand fluoroscopy‐guided approach. A single clinical case was included to examine the translational potential of the guide‐assisted method in a clinical setting. We hypothesized that percutaneous pedicle screw fixation is feasible in dogs, and that patient‐specific 3D‐printed drill guides provide superior accuracy compared to the freehand technique.

## 2 MATERIALS AND METHODS

### 2.1 Cadaveric study

#### 2.1.1 Cadaveric specimens

A total of 13 Beagle cadavers, euthanized for reasons unrelated to this study, were included (8 males, 5 females; bodyweight median 10.4 kg [interquartile range: IQR 9.4–10.8; range: 5.6–13.0]; age median 2 years [IQR 1–2]). Preoperative computed tomography (CT) confirmed no advanced degenerative change at the lumbosacral junction; one cadaver exhibited a small dorsal fracture of the caudal L7 endplate, remote from the planned pedicle pathway, and was retained. All cadavers were obtained from dogs that participated in unrelated terminal studies, which were approved by the Institutional Animal Care and Use Committee of corresponding institution (approval nos: 202304A‐XXX‐011 and 202404A‐XXX‐066). Cadavers were frozen at −20°C and thawed 24 h prior to the surgical procedures for use.

#### 2.1.2 Preoperative planning for pedicle screw insertion trajectory

CT (Aquillion prime SP, TSX‐303B, Canon Medical systems, Japan) scans of the lumbosacral junction were performed with the patient in sternal recumbency. Images were acquired with a slice thickness of 0.5 mm. The DICOM (Digital Imaging and Communications in Medicine) images were processed using the 3D Slicer version 5.6.2(Surgical Planning Lab, Brigham and Women's Hospital, Harvard Medical School, Boston, Massachusetts) to isolate the lumbosacral junction required for guide fabrication, and the segmented data were exported as stereolithography (STL) files. These STL files were further processed using 3D modeling software (3ds Max, Autodesk Inc., USA) to design patient‐specific drill guides for pedicle screw fixation.

**FIGURE 1:**
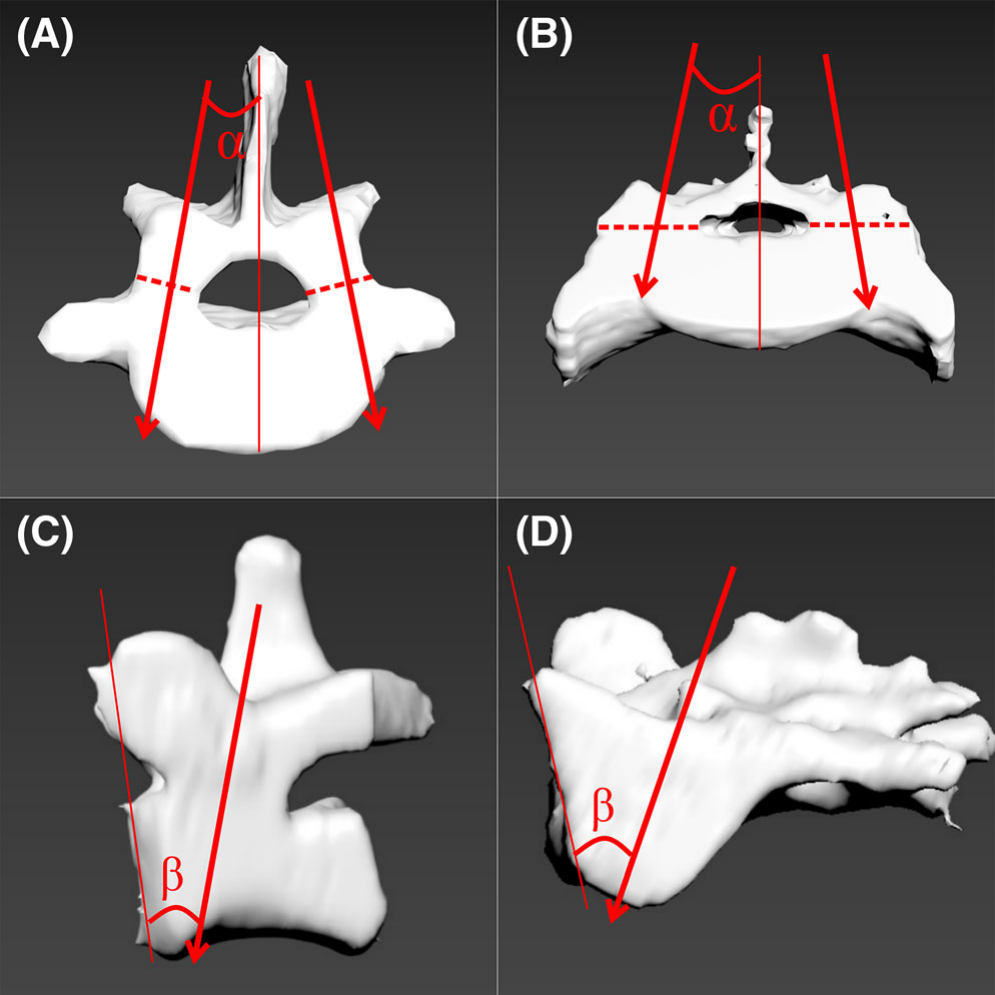
Transverse computed tomography (CT)‐based three‐dimensional (3D) models used to assess optimal pedicle screw trajectories in the canine lumbosacral junction. Transverse views of the (A) L7 and (B) S1 show planned pedicle screw trajectories (red arrows); the midline reference is indicated for transverse angular measurements, and a dashed red line denoted pedicle with measurement. Sagittal views of (C) L7 and (D1) S1 show planned trajectories (red arrows) with sagittal angles measured relative to the cranial endplate.

The transverse sections of the lumbosacral bone were carefully analyzed to identify the safe corridor for screw insertion (Figure 1). The insertion point for the L7 vertebra was determined based on previously established anatomical landmarks, specifically the caudal part of the facet joint.^9^, ^17^, ^18^ For the sacrum, the insertion point was located in the sacral fossa, just caudal to the sacral articular surface and cranial to the S1 foramen.^9^, ^17^, ^18^ Screw diameter was selected as approximately two‐thirds of the pedicle width at the insertion point, ensuring sufficient space for screw fixation without compromising adjacent neural structures.^18^ The screw depth was designed to penetrate at least 80% of the vertebral body, following human surgical standards, and bicortical fixation was incorporated to enhance stability.^9^, ^19^

To ensure accurate screw trajectory planning, both transverse and sagittal insertion angles were measured using the preoperative CT data. In the transverse plane, the angle was calculated between the screw axis and a reference line that was parallel to the spinous processes and bisected the vertebral body. In the sagittal plane, the angle was defined between the screw trajectory and the cranial endplate of each vertebra. These planned angles were used to guide fabrication and were integrated into the final 3D‐PSG design. The same planned trajectories and insertion points were also used as reference standards for the freehand technique.

Implant sizing followed the preoperative measurements and was standardized across Beagle cadavers: L7 screws 2.0 × 18 mm, S1 screws 3.5 × 18 mm invetra Pedicle Screw Fixation System (Neuromed, Huddersfield, UK).

#### 2.1.3 Design of 3D‐printed patient‐specific drill guide

**FIGURE 2:**
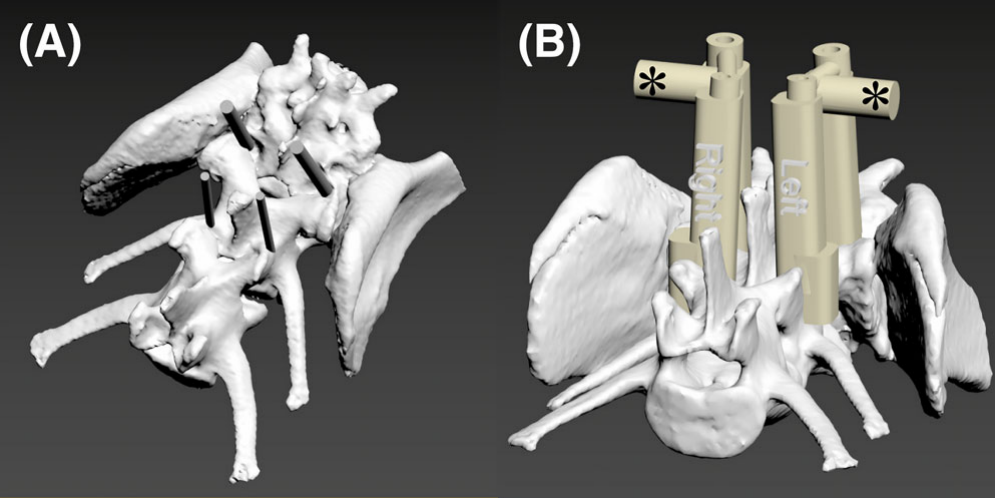
Three‐dimensional visualization of pedicle screw planning and patient‐specific drill guide design for the canine lumbosacral junction. Oblique views of the L7–S1 vertebrae showing simulated pedicle screw trajectories as (A) gray cylinders and (B) bilateral patient‐specific three‐dimensional (3D)‐printed patient‐specific drill guides (yellow) placed over the dorsal aspect of the vertebrae. The guide sleeves are aligned with the planned trajectories, and the entry points correspond to the drill bit diameter and screw insertion sites. The lateral bar (asterisk) provides additional stabilization during placement and fluoroscopic verification.

The 3D‐PSG was designed to fit the bone surface precisely, ensuring compatibility with a minimally invasive approach. For the L7 vertebra, the guide was contoured to align with the facet joint and extend to the vertebral body, providing stable fixation. Similarly, for the S1 vertebra, the guide was designed to engage with the sacral fossa and connect seamlessly with the L7 guide. The length of the guide holes was extended dorsally to ensure they protruded above the soft tissue, facilitating easy placement and alignment. Additionally, a lateral bar was incorporated into the design to maintain the guide's position securely during fluoroscopic imaging, minimizing displacement during verification (Figure 2).

The 3D‐PSG was exported as STL files and 3D printed using a resin‐based 3D printer (Photon M3 Max, Anycubic, Shenzhen, China). The printing process took approximately 3 h using resin (ZMD‐1000B CLEAR‐SG, Zenith, Daegeon, Korea). The guides were post‐processed by washing in isopropyl alcohol for 5 min and cured under UV light for 5 min to ensure proper hardening and mechanical stability.

#### 2.1.4 Surgical technique

**FIGURE 3:**
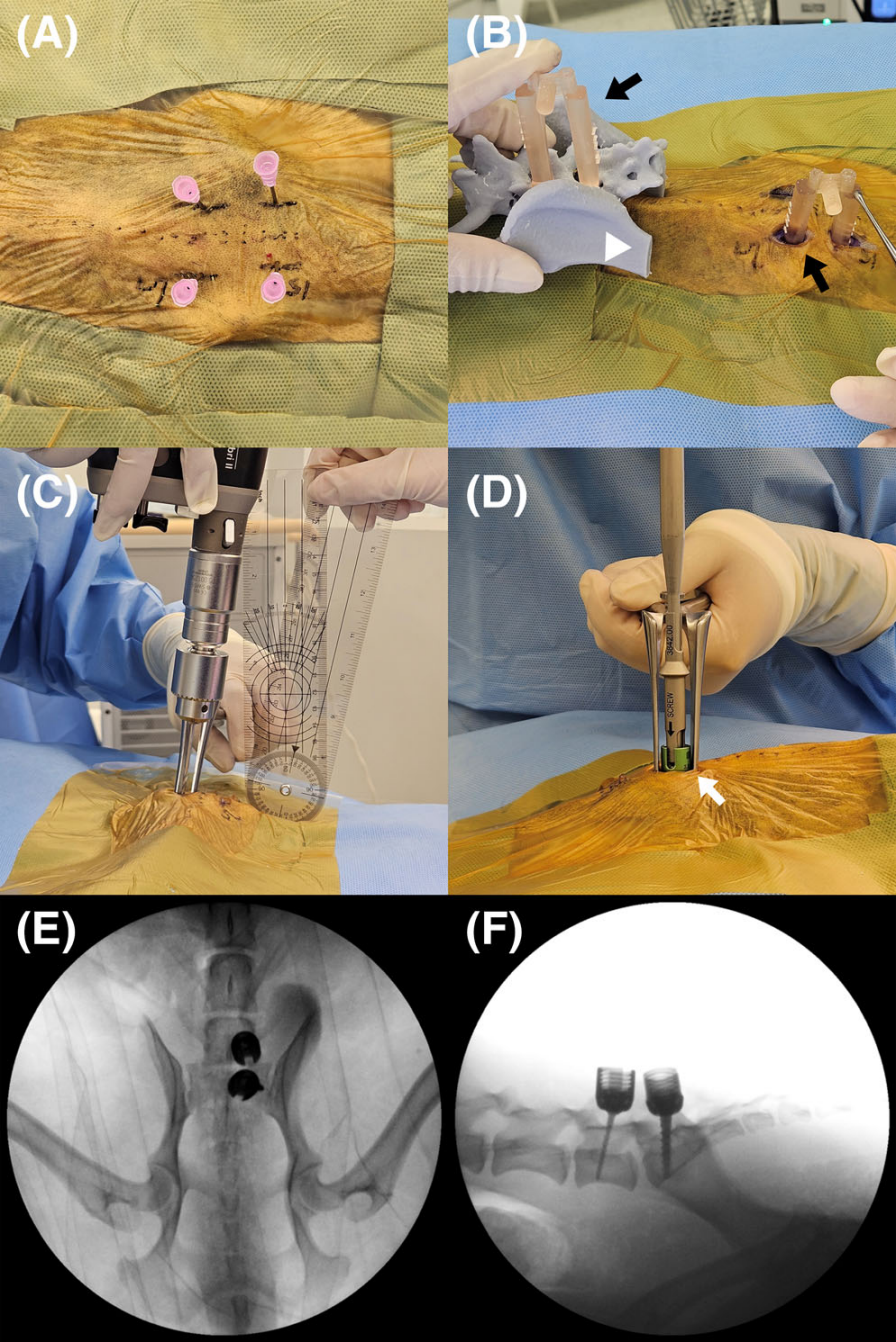
Cadaveric procedural steps for pedicle screw placement using a three‐dimensional (3D)‐printed patient‐specific drill guide (3D‐PSG) and the freehand technique. (A) Preoperative planning of insertion sites at L7 and S1 using 18 gauge spinal needles under C‐arm fluoroscopic guidance with corresponding skin markings. (B) Placement of a 3D‐PSG on the cadaveric lumbosacral region (black arrow) and a matched 3D‐printed bone model for reference (white arrow). (C) Freehand drilling performed according to the preoperative plan with intraoperative angle confirmation using a goniometer, with the same verification applied during 3D‐PSG‐assisted drilling. (D) Insertion of a 3.5‐mm pedicle screw (white arrow) into the S1 vertebra using a screwdriver and introducer sleeve while maintaining soft‐tissue retraction. (E) Anteroposterior fluoroscopic view after screw placement demonstrating trajectory relative to the pedicle and midline. (F) Lateral fluoroscopic view after screw placement demonstrating sagittal alignment relative to the cranial endplate.

Surgical procedures were performed with cadavers placed in sternal recumbency, maintaining a neutral frog‐leg position to optimize access to the lumbosacral junction. Symmetric positioning was confirmed in all specimens using anteroposterior (AP) fluoroscopy by aligning the midline spinous processes and verifying symmetry of the iliac wings. Sagittal alignment was assessed on lateral fluoroscopy using the L7 transverse process and the S1 endplate as anatomic landmarks. Under C‐arm fluoroscopic guidance, spinal needles were placed to mark the planned entry points at the caudal aspect of cranial articular process of L7 and the sacral fossa of S1, and their positions were confirmed on both AP and lateral fluoroscopic views (Figure S1). In each specimen, the left and right sides were randomly assigned to either the 3D‐PSG group or the freehand group, allowing intrasubject comparison. On the PSG‐assisted side, pedicle screw fixation was performed by a novice surgeon (author 5) without prior experience in lumbosacral stabilization surgery. The facet joints of L7 and the sacral fossa of S1 were identified under C‐arm fluoroscopic guidance, and spinal needles were used to mark the insertion points. Small skin incisions (approximately 20 mm) were made over the L7 and S1 regions, large enough to accommodate the guide sleeves. Blunt dissection was performed to expose the bony landmarks with minimal disruption to surrounding soft tissues. The 3D‐PSG was then press‐fitted onto the bony surface, engaging both the L7 facet joint and the S1 sacral fossa. The guide's trajectory corresponded with the preoperatively measured insertion angles. To ensure accurate guide seating, the guide was positioned until it securely engaged its predefined bony contact points without observable slippage. Proper seating was verified by confirming guide immobility against the cortical surface and maintaining firm bony engagement throughout drilling. Alignment of the drill trajectory relative to the spinous process was visually assessed to confirm maintenance of the intended insertion angle. In the transverse plane, the target trajectory was reproduced using a goniometer aligned with the drill axis, while sagittal alignment was verified under lateral fluoroscopy. Fluoroscopy was used intermittently during drilling and after screw placement to confirm maintenance of the planned trajectory, and all fluoroscopic checks performed for guide alignment verification were included in the total number of fluoroscopic acquisitions reported. A 1.5 mm drill bit was used for L7, followed by a 2.5 mm drill bit for S1. Screws were placed into predrilled holes, and the screw heads were intentionally left approximately 5 mm proud to facilitate subfascial rod insertion without additional soft tissue retraction (Figure 3).On the freehand side, screw fixation was performed by an experienced surgeon (author 4). The insertion points at L7 and S1 were also marked under fluoroscopy. After skin incision and soft tissue dissection, the drilling angle was manually adjusted according to the preoperative CT‐based trajectory. In the transverse plane, the desired insertion angle was reproduced using a goniometer aligned with the drill axis, while sagittal alignment was verified using lateral fluoroscopy. Fluoroscopy was used intermittently during drilling and after screw placement to confirm alignment (Figure 3). A preliminary hole was created with a burr or K‐wire to prevent drill slippage. Screws of the same size and length as those used in the guide group were then inserted.In both techniques, the musculature between L7 and S1 was retracted using a vaginoscope to preserve muscle integrity. A titanium rod was inserted in a cranio‐caudal direction and seated into the pedicle screw heads without creating additional rod‐dedicated skin incisions or connecting the skin incision. The rod was secured using locking set screws with a torque‐limiting screwdriver. Routine skin closure was performed, and postoperative CT imaging was conducted with the cadavers maintained in the same sternal recumbency position.

#### 2.1.5 Intraoperative evaluation

Operating time (min), fluoroscopic imaging frequency (acquisitions, count), and incision length (mm) were recorded to assess procedural efficiency and invasiveness. The total procedure time (min) was measured from the initial identification of anatomical landmarks at L7 and S1 under fluoroscopy to the completion of pedicle screw insertion. The procedure was divided into defined phases: insertion point identification, skin incision, soft tissue dissection, placement of the 3D‐PSG or drill angle adjustment in the freehand group, drilling, and screw placement. Radiation exposure was indirectly assessed by counting the number of fluoroscopic acquisitions (count), under otherwise standardized conditions. Incision lengths (mm) were measured at the L7 facet and S1 fossa levels.

#### 2.1.6 Postoperative evaluation

Pedicle screw placement accuracy was evaluated based on the degree of medial, lateral, cranial and caudal pedicle wall breach observed in axial CT images. Screw breaches of the pedicle cortex in the axial lane were classified into three grades according to the previous described system. Grade 0 indicates no vertebral canal breach, grade 1 indicates a breach of the pedicle wall of less than half of the screw diameter and grade 2 indicates a breach exceeding half of the screw diameter.^20^, ^21^

To evaluate the accuracy of percutaneous pedicle screw fixation, the deviation in inclination angles between preoperative planning and actual operative outcomes was assessed. This angle deviation was quantified and compared between the 3D‐PSG group and the freehand group, with the objective of identifying the group with the smaller inclination angle deviation. orientation in each plane.

#### 2.1.7 Statistical analysis

A priori power analysis was performed using G*Power (version 3.1; Heinrich Heine University, Düsseldorf, Germany) to determine the minimum required sample size. Based on an effect size of 1.0, α = 0.05, and power (1–β) = 0.95, a total sample size of 13 cadavers was calculated. Statistical analyses were performed using SPSS version 26.0 (IBM Corp., Armonk, New York). Continuous variables were tested for normality using the Shapiro–Wilk test. Normally distributed variables were compared using the paired *t*‐test and presented as mean ± SD. Non‐normally distributed or discrete variables were compared using the Wilcoxon signed‐rank test and presented as median and interquartile range (IQR). Categorical data, such as vertebral canal breach grades, were analyzed using Fisher's exact test. For this analysis, any breach (grade ≥1) was treated as “present,” whereas grade 0 was considered “absent,” acknowledging that grade 1 breaches were regarded as clinically insignificant. Multiple outcome measures within each vertebral level were treated as independent exploratory analyses without formal adjustment for multiple comparisons. A *p*‐value <.05 was considered statistically significant.

### 2.2 Case report

A 5‐year‐old castrated male German Shepherd Dog, weighing 30 kg, was referred for evaluation of a three‐year history of progressive lumbosacral pain with intermittent hindlimb lameness that waxing and waning depending on medical treatment. Although the lameness initially responded to conservative therapy, clinical signs had recently worsened and became refractory to medication. Orthopedic examination revealed no clinically significant abnormalities. Neurologic examination revealed pain upon palpation of the lumbosacral (LS) junction, marked pain on lordosis testing, and discomfort during tail elevation.

General anesthesia was administered to facilitate both magnetic resonance imaging (MRI) and CT imaging for diagnostic evaluation and surgical planning. Premedication included intramuscular midazolam (1 mg/kg), followed by intravenous induction with propofol (4 mg/kg), and anesthesia was maintained using isoflurane in oxygen via endotracheal intubation. MRI was performed in both flexion and extension positions of the lumbosacral spine to assess dynamic motion‐dependent compression. Imaging in the extended position revealed features consistent with degenerative lumbosacral stenosis (DLSS) at the L7–S1 junction, including telescoping of the sacral lamina and mild dorsal bulging of the intervertebral disc. These findings were notably alleviated in the flexed position, suggesting dynamic compression. Subsequently, CT was performed for 3D‐PSG fabrication. To replicate the expected intraoperative posture and ensure optimal guide fit, CT was acquired with the hindlimbs positioned in flexion, mimicking the surgical setup.

**FIGURE 4:**
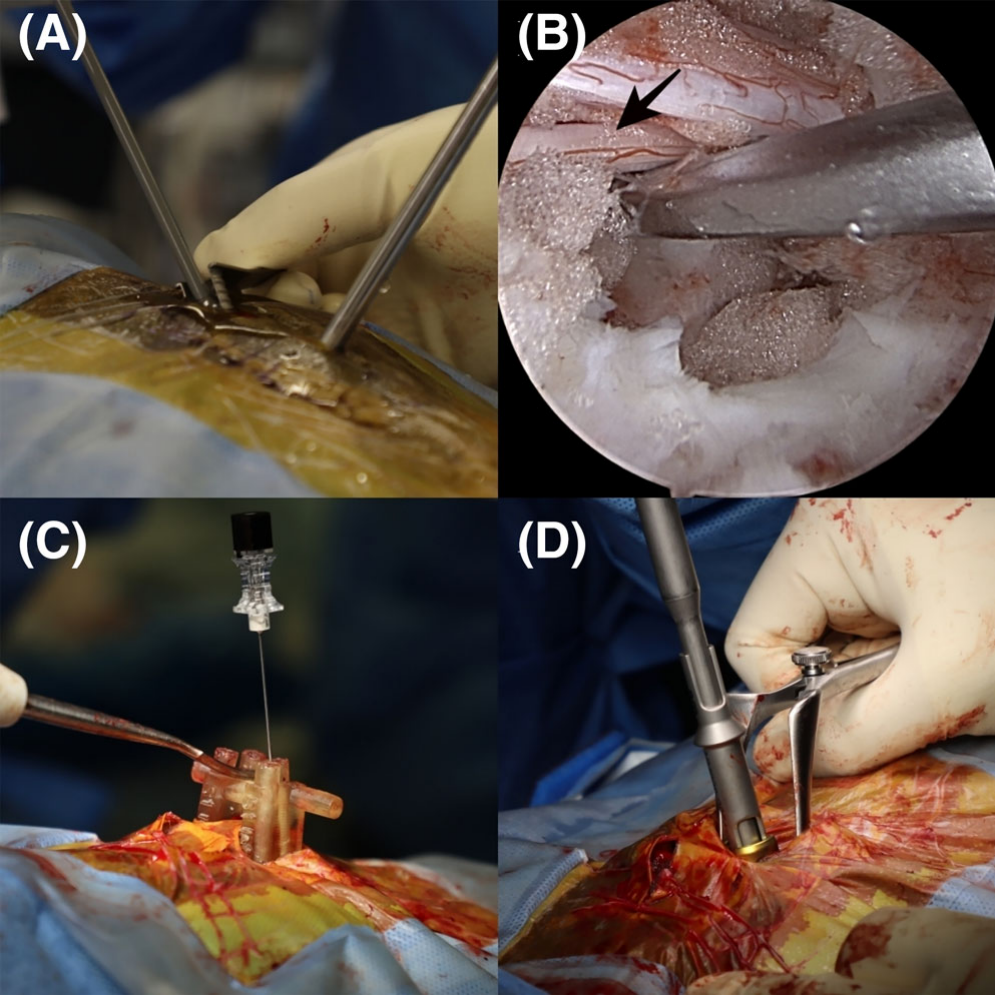
(A) A unilateral biportal endoscopic approach was applied for dorsal laminectomy and discectomy, with creation of a viewing portal and a working portal. (B) Endoscopic view of the lumbosacral junction showing the caudal equina retracted by a probe (black arrow). (C) A three‐dimensional (3D)‐printed patient‐specific drill guide was placed on the sacral region, aligned with the preoperative computed tomography (CT)‐based trajectory. (D) Pedicle screws were inserted into the S1 and S2 vertebrae using a screwdriver and introducer sleeve, following soft tissue retraction with vaginoscope.

**FIGURE 5:**
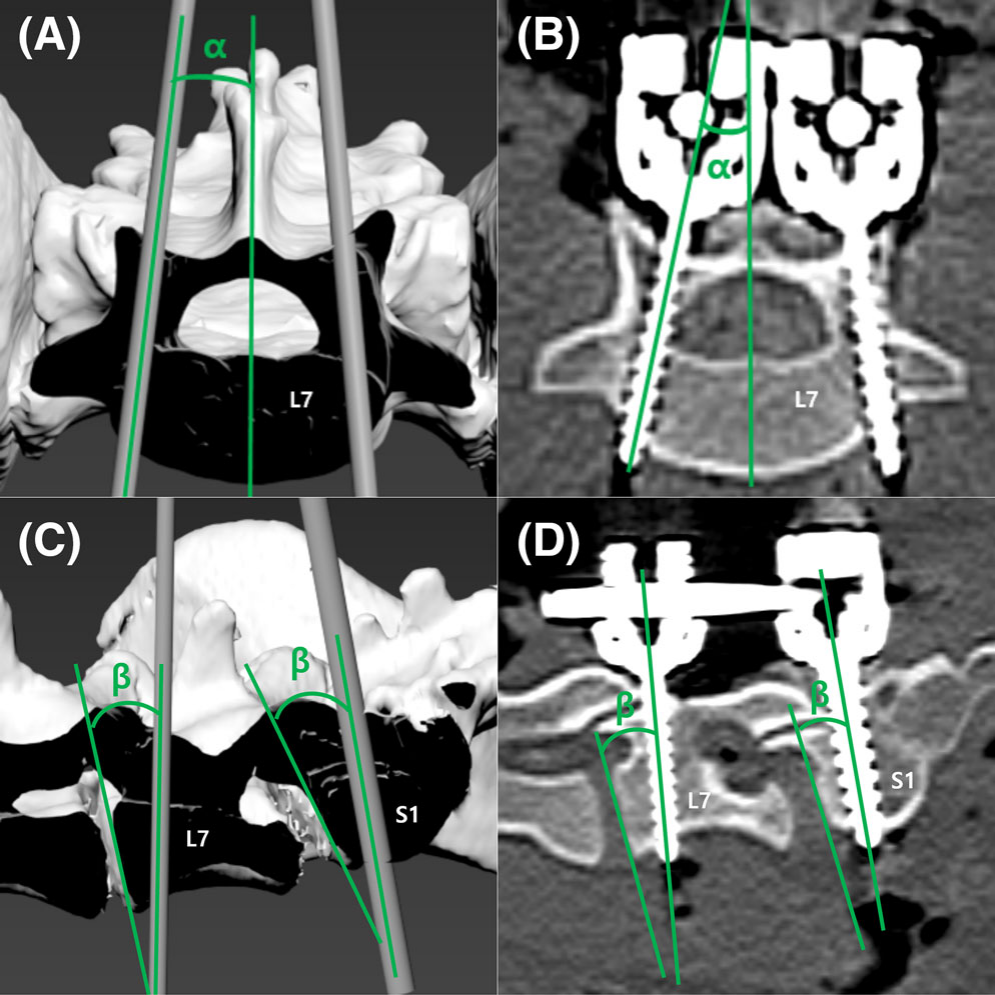
Assessment of transverse and sagittal angular deviation for pedicle screw placement. Transverse plane images of the L7 vertebra showing the planned transverse angle (α) on (A) the preoperative three‐dimensional (3D) model and (B) the actual angle on the postoperative computed tomography (CT) image. Angle α was defined as the angle between the sagittal plane and a line along the screw axis. Sagittal plane views showing the planned sagittal angle (β) on (C) the preoperative 3D models of L7 and S1 and (D) the actual screw positions on postoperative sagittal CT reconstructions. Angle β was defined as the angle between a line along the cranial vertebral endplate and the screw axis in the sagittal plane. Angular deviation was determined by comparing preoperative plans with postoperative screw.

The procedure began with dorsal laminectomy and discectomy using a Unilateral biportal endoscopy (UBE) technique to decompress the L7–S1 intervertebral space (Figure 4).^22^ Based on preoperative CT, the medial border of the pedicle (LMBP) was mapped perpendicular to the midline; two points along the LMBP were marked and connected, and cranial and caudal portal sites were established relative to the midpoint. After blunt dissection of soft tissues form the bony surface, continuous irrigation was established through a cannula, and the obturator was exchanged for a 4.0 mm 0° rigid endoscope (SPINUSS SCOPE, Endovision). A radiofrequency device was used to detach loosely adherent soft tissues and improve visualization. Dorsal laminectomy was performed using a 3 mm hooded burr (AR‐9300RBT, Arthrex, Naples, Florida) and Kerrison rongeur (Solendos, Seoul, Republic of Korea) to the preplanned extent, then extended until the bilateral L7 and S1 nerve roots were identified and probed. Discectomy was subsequently conducted by retracting the cauda equina and removing disc material with graspers and a shaver (Video clip S1). Following decompression, the 3D‐PSG were applied bilaterally. On the UBE side, the L7 guide was inserted through the cranial portal and the S1 guide through the caudal portal, whereas on the contralateral side, guides were applied via separate small stab incisions. The patient was placed in sternal recumbency with the hindlimbs in a frog‐leg posture, ensuring the LS junction was maintained in slight flexion. The intervertebral disc space was not surgically distracted, and the lumbosacral junction was stabilized in slight flexion. Unlike the cadaveric protocol, fixation was extended to include the S2 vertebra, and bilateral guides were designed separately: one for both sides of L7 and another spanning S1 and S2. The facet joints were identified under fluoroscopic guidance, and small incisions were made for guide or drill bit placement. A 2.5 mm drill bit was used at L7 and a 3.2 mm bit at S1 and S2. Based on CT planning, pedicle screws from the invetra Pedicle Screw Fixation System (Neuromed) were inserted: 3.5 × 25 mm in L7, 4.5 × 25 mm in S1, and 4.5 × 15 mm in S2 (Figure 4). After elevating the intervertebral musculature, a connecting rod was placed between L7 and S1–S2. The skin was closed routinely, and a postoperative CT scan was obtained in the same position (Figure 5).

## 3 RESULTS

### 3.1 Cadaveric study

#### 3.1.1 Intraoperative evaluation

The total operation time did not differ significantly between the 3D‐PSG group (22.65 ± 2.71 min) and the freehand group (24.82 ± 5.00 min) (*p* = .139). The number of radiation frequencies acquired was significantly lower in the 3D‐PSG group (median: 5; IQR: 5–8) compared with the freehand group (9; IQR: 7–14) (*p* = .003) (Table 1).

**TABLE 1:** Intraoperative parameters in the 3D‐PSG guide and freehand groups (*n* = 13).

Item	3D‐PSG group	Freehand group	*p*‐value
Operation time (min)	22.65 ± 2.71	24.82 ± 5.00	.139
L7 incision length (mm)	17.77 ± 3.47	14.77 ± 2.89	.043^a^
S1 incision length (mm)	17.46 ± 4.82	14.23 ± 3.49	.038^a^
Radiation frequency	5 (5–8)	9 (7–14)	.003^a^

*Note*: Normally distributed continuous variables were analyzed using paired *t*‐tests, whereas radiation frequency, treated as count data, was analyzed using the Wilcoxon signed‐rank test.

Abbreviation: 3D‐PSG, three‐dimensional printed patient‐specific drill guide.

^a^ *p* < .05. *p*‐values indicate statistical comparisons between the 3D‐PSG and freehand groups for each intraoperative parameter.

The incision length at L7 was significantly greater in the 3D‐PSG group (17.77 ± 3.47 mm) than in the freehand group (14.77 ± 2.89 mm) (*p* = .043).

At S1, the 3D‐PSG group also showed a significantly longer incision length (18.0 mm; IQR: 13.0–19.0) compared with the freehand group (14.23 ± 3.49 mm) (*p* = .038) (Table 1).

#### 3.1.2 Vertebral canal breach

A total of 52 pedicle screws were inserted in 13 cadaveric specimens, with 26 screws placed using a unilateral 3D‐PSG and 26 screws inserted using a contralateral freehand technique within the same specimens. The 3D‐PSG group was operated by a novice surgeon without prior experience in lumbosacral stabilization, while the freehand group procedures were performed by an experienced surgeon.

The distributions of pedicle wall breach grades and corresponding *p*‐values are summarized in Table 2. Medial and cranial pedicle wall breaches were identified and graded on postoperative CT images. All lateral and caudal cortices remained intact (grade 0) in both groups, precluding statistical comparison for these directions.

**TABLE 2:** Grade of vertebral breach.^a^

Vertebrae	Direction	3D‐PSG group	Freehand group	*p*‐value
L7, *n* = 13	Medial	Grade 0: 12 Grade 1: 0 Grade 2: 1	Grade 0: 7 Grade 1: 2 Grade 2: 4	.073
	Lateral	Grade 0: 13 Grade 1: 0 Grade 2: 0	Grade 0: 13 Grade 1: 0 Grade 2: 0	N/A^b^
	Cranial	Grade 0: 13 Grade 1: 0 Grade 2: 0	Grade 0: 13 Grade 1: 0 Grade 2: 0	N/A^b^
	Caudal	Grade 0: 13 Grade 1: 0 Grade 2: 0	Grade 0: 13 Grade 1: 0 Grade 2: 0	N/A^b^
S1, *n* = 13	Medial	Grade 0: 10 Grade 1: 2 Grade 2: 1	Grade 0: 11 Grade 1: 1 Grade 2: 1	1.000
	Lateral	Grade 0: 13 Grade 1: 0 Grade 2: 0	Grade 0: 13 Grade 1: 0 Grade 2: 0	N/A^b^
	Cranial	Grade 0: 11 Grade 1: 2 Grade 2: 0	Grade 0: 13 Grade 1: 0 Grade 2: 0	0.48
	Caudal	Grade 0: 13 Grade 1: 0 Grade 2: 0	Grade 0: 13 Grade 1: 0 Grade 2: 0	N/A^b^

Abbreviation: 3D‐PSG, three‐dimensional printed patient‐specific drill guide; N/A, not applicable.

^a^ Grade 0: no breach; Grade 1: <50% breach of the screw diameter; Grade 2: ≥50% breach of the screw diameter.

^b^ N/A: no breaches observed or no statistical comparison possible due to zero variance.

At the L7 vertebra, the 3D‐PSG group exhibited 12 screws classified as grade 0, 0 as grade 1, and one as grade 2, whereas the freehand group showed seven, two, and four screws in grades 0, 1, and 2, respectively. The distribution of vertebral canal breach grades did not differ significantly between groups at this level (*p* = .073).

At the S1 vertebra, the 3D‐PSG group showed 10 screws in grade 0, two in grade 1, and one in grade 2. The freehand group had 11, one, and one screw in each grade, respectively. No statistically significant difference was noted at the S1 level (*p* = 1.000).

Regarding cranial breaches, no cranial pedicle breaches were identified in L7 in either group. At the S1 level, two screws in the 3D‐PSG group were classified as grade 2, while all remaining screws were grade 0. Due to the absence of cranial breaches in the freehand group, statistical comparison was not possible.

#### 3.1.3 Pedicle screw angle deviation

Transverse angle deviation at the L7 vertebra was significantly smaller in the 3D‐PSG group (6.4 ± 4.14°) than in the freehand group (12.8 ± 8.58°) (*p* = .036). No significant difference was observed at S1 between the 3D‐PSG (6.22 ± 4.05°) and freehand (7.62 ± 7.82°) groups (*p* = .624) (Table 3).

**TABLE 3:** Transverse angle deviation in the 3D‐PSG and freehand groups (mean ± SD, *n* = 13).

Vertebrae	3D‐PSG group	Freehand group	*p*‐value
L7 (degrees)	6.4 ± 4.14	12.8 ± 8.58	.036^a^
S1 (degrees)	6.22 ± 4.05	7.62 ± 7.82	.624

Abbreviation: 3D‐PSG, three‐dimensional printed patient‐specific drill guide.

^a^ *p* < .05. Transverse angle deviations were compared between the 3D‐PSG and freehand groups at L7 and S1 levels.

Sagittal angle deviation was also significantly lower in the 3D‐PSG group at L7 (median: 2.7 IQR: 1.5–4.75°) compared with the freehand group (5.82 ± 3.01°) (*p* = .018). At S1, the 3D‐PSG (3.05 ± 2.39°) and freehand (median: 2.6 IQR: 0.95–4.05°) groups did not differ significantly (*p* = .625) (Table 4).

**TABLE 4:** Sagittal angle deviation in the 3D‐PSG and freehand groups (*n* = 13).

Vertebrae	3D‐PSG group	Freehand group	*p*‐value
L7 (degrees)	2.58 ± 2.42	5.82 ± 3.01	.018^a^
S1 (degrees)	2.7 (1.5–4.75)	2.6 (0.95–4.05)	.625

*Note*: Normally distributed continuous variables were analyzed using paired *t*‐tests (mean ± SD), whereas radiation frequency was analyzed using the Wilcoxon signed‐rank test (median [IQR]).

Abbreviations: 3D‐PSG, three‐dimensional printed patient‐specific drill guide; IQR, interquartile range.

^a^ *p* < .05. Sagittal angle deviations were compared between the 3D‐PSG and freehand techniques at L7 and S1.

### 3.2 Case outcome

**FIGURE 6:**
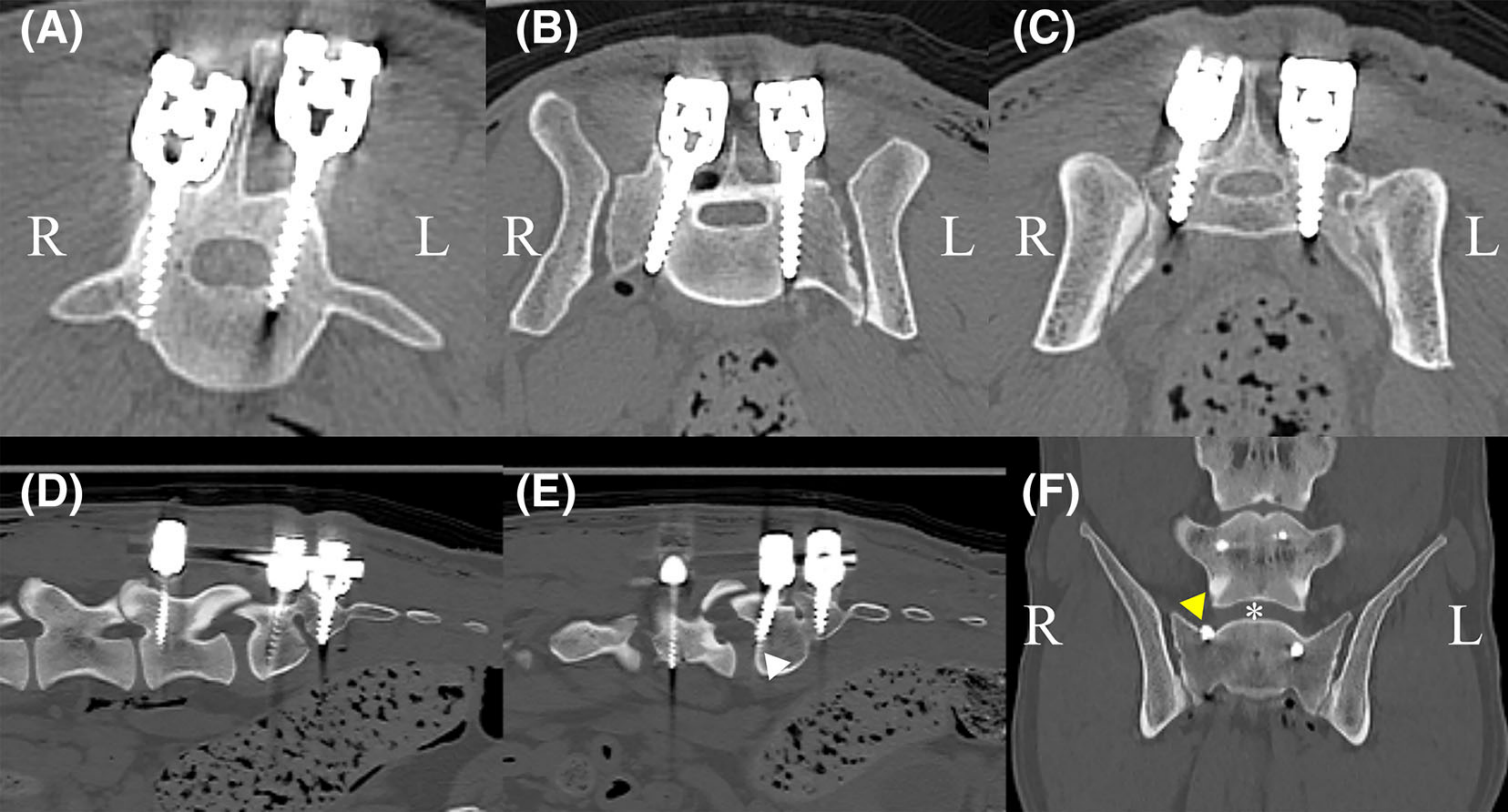
Postoperative computed tomography (CT) images of the clinical case. Postoperative CT images of the clinical case demonstrating pedicle screw positions at L7, S1, and S2. Transverse views (A–C), sagittal views (D, left; E, right), and a dorsal view (F) are shown. (A) The L7–left pedicle screw exhibits a grade 1 medial canal breach. (E) The S1 pedicle screw traverses the cranial endplate (white arrowhead), indicating a cranial breach grade 2. (F) There is no intradiscal penetration; the disc is marked by an asterisk, and a mild scuffing injury is visible along the lateral aspect of the disc (yellow arrowhead). L, left; R, right.

The procedure was completed without intraoperative complications. Case outcomes were quantified: operative time 60 min (UBE) + 40 min (PPSF); fluoroscopic acquisitions 6 (UBE) and 10 (PPSF). A medial canal breach was classified as grade 1 at L7 left (Figure 6), with grade 0 elsewhere. An additional cranial breach grade 2 at S1‐right was identified. On dorsal projection, there was no intradiscal penetration, with only a mild scuffing injury along the lateral aspect of the disc. Detailed angular deviations and breach annotations are provided in Table S1. The intervertebral foraminal area increased bilaterally: from 0.102 to 0.160 cm^2^ on the right side, and from 0.116 to 0.183 cm^2^ on the left side. The mid‐portion disc space height, measured according to the method described previously,^23^ increased from 4.14 mm preoperatively to 5.26 mm postoperatively.

Clinically, the patient exhibited improved tolerance to sternal recumbency and a reduced pain response to lumbosacral palpation within 24 h after surgery. No neurologic deterioration was observed, and the patient was discharged the following day. At the 3‐month recheck, the patient showed improved gait and maintained neurologic stability without evidence of symptom recurrence (Video clip S2).

## 4 DISCUSSION

This study aimed to evaluate the feasibility and accuracy of PPSF in the canine lumbosacral junction using 3D‐PSG. The proposed hypotheses were that PPSF would be feasible in cadaveric canine models, and that the use of 3D‐PSG would improve accuracy relative to the freehand technique. The findings of this study partially support these hypotheses. The 3D‐PSG technique demonstrated high accuracy and safety at L7, with reduced angular deviation and no canal breach, whereas no significant difference was observed at S1. These results suggest that the benefits of the guide may be more pronounced in anatomically constrained regions.

Although the difference in vertebral canal breach rates at L7 did not reach statistical significance (*p* = .073), a trend toward fewer breaches was observed in the 3D‐PSG group (1/13) compared to the freehand group (6/13). This difference is likely attributable to the narrower pedicle corridor and higher risk of cortical breach at L7.^9^ Similarly, no statistically significant difference in breach rates was observed at the S1 level, likely due to the wider anatomic corridor of the sacral pedicle.^9^ However, transverse angle deviation was significantly lower in the 3D‐PSG group, particularly at L7, suggesting that patient‐specific guidance may provide greater benefit where anatomic constraints are most pronounced. Sagittal angle deviation was also reduced in the 3D‐PSG group. These findings emphasize the utility of 3D‐PSG in achieving accurate screw placement, particularly in technically demanding segments such as L7.

While the freehand technique involved shorter skin incisions (15.0 mm) compared to the 3D‐PSG approach (18.0 mm), this difference was likely due to the structural requirements of the 3D‐PSG. Despite the slightly longer incision, the 3D‐PSG group showed superior outcomes in both accuracy and prevention of canal breaches. The higher incidence of vertebral canal breaches in the freehand group may be explained by technical limitations inherent to the approach. One contributing factor was the lack of intraoperative visualization of the transverse angulation, which required the surgeon to rely on external estimation using a goniometer. This method introduced the potential for operator‐dependent errors. In addition, the restricted visual field associated with the minimally invasive approach limited the ability to identify and align with anatomical landmarks such as the spinous process and lamina.^24^, ^25^ Another factor that may have affected screw placement accuracy was the use of a non‐cannulated drill system, which differs from the cannulated systems commonly employed in human spinal procedures. In contrast, the guide‐assisted technique facilitated consistent screw placement with minimal reliance on fluoroscopic confirmation. As a result, radiation exposure was significantly reduced in the 3D‐PSG group, enhancing safety for both the surgeon and the patient, and contributing to a more efficient surgical workflow.

In our study, the anatomical characteristics of the lumbosacral junction influenced guide design and stability. While L7 provided stable fixation of the guide via the facet joint, guide anchoring at S1 was less secure due to limited contact area and proximity to the iliac wings. In some cases, contact was further interfered with by the prominence of the intermediate sacral crest, and the concave morphology of the sacral fossa interfered with stable seating of the guide on the dorsal surface of S1. For this reason, the 3D‐PSG was designed as a single unilateral component spanning L7 to S1, thereby preserving sagittal alignment between preoperative CT and intraoperative posture. To improve transverse angle alignment, a box‐shaped structure was incorporated into the guide to envelop the L7 facet, allowing more consistent rotational positioning. As a result, the incidence of canal breach was significantly lower in the guide group compared to the freehand group. Nevertheless, due to the unilateral design, sagittal angle deviations were generally smaller than transverse deviations in the 3D‐PSG group, suggesting that sagittal alignment was better preserved. It is notable that bilateral guide placement was not feasible at S1 alone due to limited bone surface area. However, in the clinical application phase, the fixation was extended to S2, thereby providing sufficient bony contact for bilateral guide stabilization. Additionally, S2 screw fixation was selected to optimize construct stability, informed by human spine literature indicating that additional sacral purchase can enhance stability.^26^ This modification enhanced transverse alignment and intraoperative stability.

The clinical case illustrates the translational applicability of the cadaveric technique. Pedicle screws were placed at L7, S1, and S2 with only minor breaches identified: a grade 1 medial canal breach at L7 left side and a grade 2 cranial breach at S1 right that traversed the cranial endplate without intradiscal penetration. Angular deviations in this case were modestly greater than the cadaveric medians, a difference that is plausible explained by the greater osseous depth and width in a large‐breed dog (providing ample purchase but amplifying small angular offsets over a long path) and by minor clearance between the guide sleeve and drill bit, which can accumulate as depth increase. Despite these offsets, bone stock was sufficient to maintain secure purchase. Postoperative CT demonstrated increased intervertebral foraminal area and disc space height compared to the preoperative extended position. However, this finding is attributable to the flexed positioning in which the lumbosacral junction was stabilized rather than surgical distraction, consistent with previous studies demonstrating that spinal flexion increases foraminal dimensions in dogs with lumbosacral disease.^27^ Taken together, these observations support that 3D‐PSG can be applied reliably in a clinical case, with small, context‐dependent trajectory offsets remaining clinically acceptable when canal violation is avoided and construct stability is preserved.

Several limitations of this study should be considered when interpreting the results. First, this study was not randomized or crossover. A relatively novice surgeon performed 3D‐PSG procedures, whereas an experienced surgeon performed the free‐hand procedures. This operator assignment inevitably introduces intersurgeon confounding and precludes definitive head‐to‐head comparison between techniques. Accordingly, any between‐technique differences should be interpreted as exploratory findings. Future studies, including multiple surgeons performing both guided and freehand procedures under controlled conditions are warranted to confirm the reproducibility of these results. Second, the cadaveric model does not fully mimic the bony characteristics of dogs with degenerative lumbosacral stenosis. As such, guide fit and screw trajectory accuracy may differ in clinical cases. In particular, actual patients with DLSS often exhibit more pronounced osteophytic changes due to chronic motion‐dependent compression, which were not represented in the cadaveric specimens. Third, implant size selection was constrained by the available screw diameters of the implant system (2.0, 2.7, and 3.5 mm). Based on preoperative CT measurements, the mean L7 pedicle width was 3.63 mm (range: 3.13–3.98 mm), and application of the selection criterion of approximately two‐thirds of the pedicle width indicated that intermediate screw sizes (2.4 mm) would have been optimal for some specimens but were not available. Consequently, a 2.0 mm screw was selected as a conservative approach to prioritize safe placement. This conservative sizing strategy may have contributed to the observed accuracy and should be considered when interpreting the results, as it may limit generalizability to clinical settings in which a wider range of screw diameters or larger implants are routinely used. Fourth, all cadaveric testing was performed using skeletally mature Beagle dogs, which may not reflect the anatomical variability or disease presentation commonly observed in breeds predisposed to DLSS; this may limit generalizability. Fifth, clinical validation was limited to a single patient, which precludes definitive conclusions regarding generalizability or long‐term outcomes. Larger prospective studies with extended follow‐up are warranted to assess the safety and accuracy of percutaneous pedicle screw fixation, implant longevity, complication rates, and broader clinical applicability across diverse patient populations. Finally, multiple outcome measures within each vertebral level were analyzed without formal adjustment for multiple comparisons; therefore, statistical significance should be interpreted with caution, and these findings should be considered exploratory.

In conclusion, this study demonstrated that 3D‐PSG enables accurate PPSF in the canine lumbosacral junction. The guide‐assisted technique reduced radiation exposure and improved screw trajectory accuracy compared to the freehand method. Although the study was limited by the use of a cadaveric model and a single clinical case, the findings support the feasibility and translational potential of 3D guide‐assisted PPSF. Further clinical studies are warranted to validate long‐term outcomes.

## AUTHOR CONTRIBUTIONS

Yeom KH, DVM: Identified suitable cases, recorded demographic information, compiled all data, interpreted data, and drafted and revised the manuscript. Yoon JP, DVM: Contributed to the design of the study, performed radiographic measurements, and interpreted data. Jeon YJ, DVM, PhD: Contributed to the design of the study, analyzed data for statistical significance and in‐line editing of the manuscript. Lee HB, DVM, PhD: Contributed to the design of the study, responsible for the surgical management of the cases, oversaw data collection, provided intraoperative photographs, interpreted data, and provided scientific, in‐line editing of the manuscript. Jeong JM, DVM, PhD: Contributed to the design of the study, responsible for the surgical management of the cases, oversaw data collection, interpreted data, and critically revised the manuscript.

All authors provided a critical review of the manuscript and endorse the final version. All authors are aware of their respective contributions and have confidence in the integrity of all contributions.

## CONFLICT OF INTEREST STATEMENT

The authors declare no conflicts of interest related to this study.

## Supporting information


**Figure S1.** Intraoperative fluoroscopy for positioning and spinal needle placement. (A) AP view before needle placement showing midline spinous process line (red line) and iliac wings (asterisks) to confirm symmetric positioning.
(B) Lateral (sagittal) view before needle placement; red arrowhead indicates the L7 transverse process and yellow arrowhead indicates the S1 endplate, used to verify sagittal alignment. (C) AP view after needle placement; red arrow marks the L7 spinal needle and yellow arrow marks the S1 spinal needle. (D) Lateral view after needle placement; red arrow marks the L7 spinal needle and yellow arrow marks the S1 spinal needle. AP, anteroposterior; L7, seventh lumbar vertebrae; S1, first sacral vertebrae.
**Figure S2.** Postoperative and 3‐month follow‐up radiographs. (A–B) Immediately postoperative: sagittal and dorsal vies. (C–D) 3‐month recheck: sagittal and dorsal views. Implant position and alignment are maintained without displacement.

**Table S1.** CT‐based angular deviations and breach annotations (clinical case).
**Video Clip S1.** Intraoperative Endoscopic Record in a Clinical Case.
This material is available as part of the online article from: *link* This supplementary video is edited using VLLO version 13.6.0 (Vismosoft, Seoul, Republic of Korea).
**Video Clip S2.** Preoperative and 3‐month Postoperative Gait Evaluation in a Clinical Case. This material is available as part of the online article from: *link* This supplementary video is edited using VLLO version 13.6.0 (Vismosoft, Seoul, Republic of Korea).
