# Supplementary figures and images for: 3D printed patient‐specific drill guide for percutaneous pedicle screw fixation in lumbosacral vertebrae in dogs: A cadaveric study and clinical case report
Source: Vet Surg. 2026 Apr 28;55(5):908–19. doi: 10.1111/vsu.70115 (PMC13360313; doi:10.1111/vsu.70115)

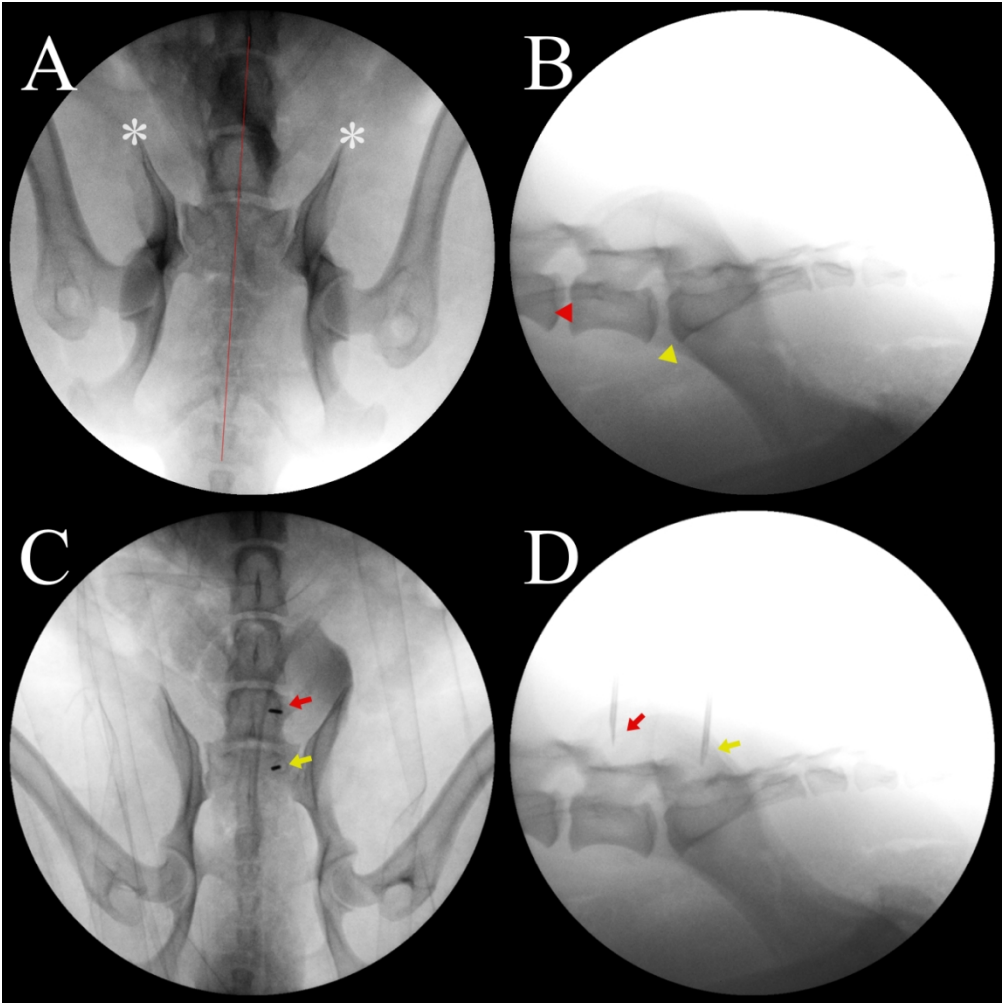

111x111mm (300 x 300 DPI)

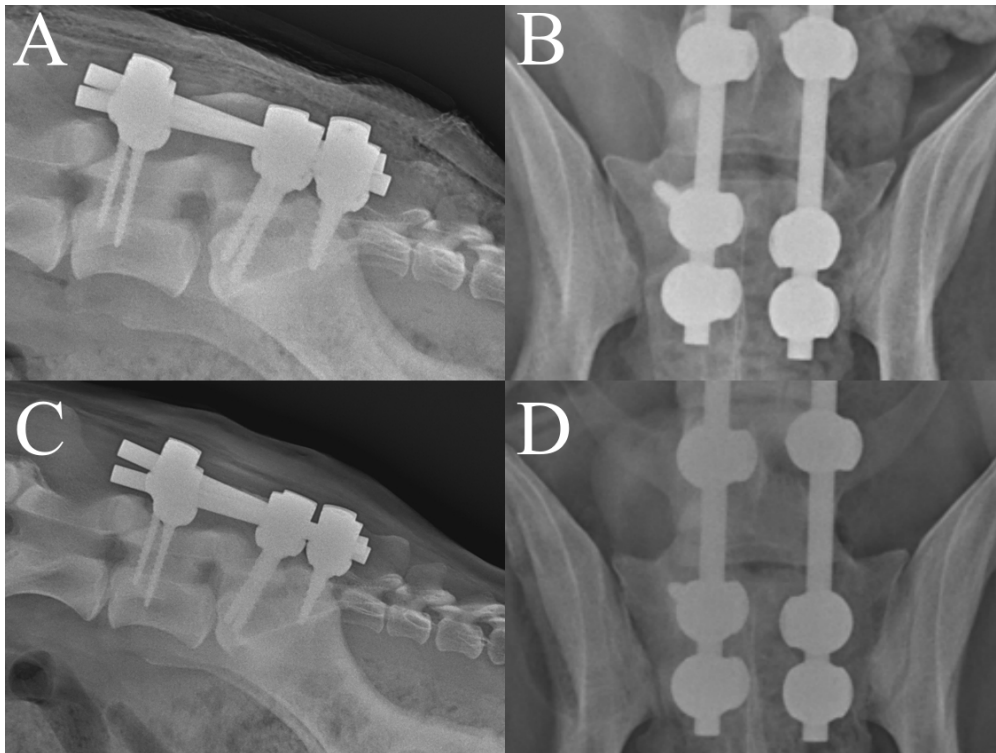

95x71mm (300 x 300 DPI)

Supplement: Supplementary file 1 — Figure S1. Intraoperative fluoroscopy for positioning and spinal needle placement. (A) AP view before needle placement showing midline spinous process line (red line) and iliac wings (asterisks) to confirm symmetric positioning. (B) Lateral (sagittal) view before needle placement; red arrowhead indicates the L7 transverse process and yellow arrowhead indicates the S1 endplate, used to verify sagittal alignment. (C) AP view after needle placement; red arrow marks the L7 spinal needle and yellow arrow marks the S1 spinal needle. (D) Lateral view after needle placement; red arrow marks the L7 spinal needle and yellow arrow marks the S1 spinal needle. AP, anteroposterior; L7, seventh lumbar vertebrae; S1, first sacral vertebrae. Figure S2. Postoperative and 3‐month follow‐up radiographs. (A–B) Immediately postoperative: sagittal and dorsal vies. (C–D) 3‐month recheck: sagittal and dorsal views. Implant position and alignment are maintained without displacement. [file VSU-55-908-s002.pdf]
