# Supplementary material for: 3D printed patient‐specific drill guide for percutaneous pedicle screw fixation in lumbosacral vertebrae in dogs: A cadaveric study and clinical case report
Source: Vet Surg. 2026 Apr 28;55(5):908–19. doi: 10.1111/vsu.70115 (PMC13360313; doi:10.1111/vsu.70115)
Supplement: Supplementary file 2 — Table S1. CT‐based angular deviations and breach annotations (clinical case). Video Clip S1. Intraoperative Endoscopic Record in a Clinical Case. This material is available as part of the online article from: link This supplementary video is edited using VLLO version 13.6.0 (Vismosoft, Seoul, Republic of Korea). Video Clip S2. Preoperative and 3‐month Postoperative Gait Evaluation in a Clinical Case. This material is available as part of the online article from: link This supplementary video is edited using VLLO version 13.6.0 (Vismosoft, Seoul, Republic of Korea). [file VSU-55-908-s001.docx]

**Supplementary Materials**

**Supplementary Table 1.** CT-based angular deviations and breach annotations (Clinical case)

| Vertebrae | Side | Transverse deviation, ° | Sagittal deviation, ° | Medial breach grade (0/1/2) | Other breach grade(0/1/2) |
| --- | --- | --- | --- | --- | --- |
| L7 | Left | 6.3 | 2.4 | 1 | 0 |
| L7 | Right | 7.2 | 3.0 | 0 | 0 |
| S1 | Left | 8.2 | 2.3 | 0 | 0 |
| S1 | Right | 7.3 | 10.9 | 0 | Cranial grade 2 |
| S2 | Left | 9.8 | 6.0 | 0 | 0 |
| S2 | Right | 4.5 | 8.8 | 0 | 0 |

**Supplementary Figure 1.** Intraoperative fluoroscopy for positioning and spinal needle placement.

(A) AP view before needle placement showing midline spinous process line (red line) and iliac wings (asterisks) to confirm symmetric positioning.

(B) Lateral (sagittal) view before needle placement; red arrowhead indicates the L7 transverse process and yellow arrowhead indicates the S1 endplate, used to verify sagittal alignment.

(C) AP view after needle placement; red arrow marks the L7 spinal needle and yellow arrow marks the S1 spinal needle.

(D) Lateral view after needle placement; red arrow marks the L7 spinal needle and yellow arrow marks the S1 spinal needle.

AP, anteroposterior; L7, seventh lumbar vertebrae; S1, first sacral vertebrae.

**Supplementary Figure 2.** Postoperative and 3-month follow-up radiographs

(A-B) Immediately postoperative: sagittal and dorsal vies. (C-D) 3-month recheck: sagittal and dorsal views. Implant position and alignment are maintained without displacement.

**Supplementary Video Clip S1.** Intraoperative Endoscopic Record in a Clinical Case

This material is available as part of the online article from: *link*

This supplementary video is edited using VLLO version 13.6.0 (Vismosoft, Seoul, Republic of Korea)

**Supplementary** **Video Clip S2.** Preoperative and 3-month Postoperative Gait Evaluation in a Clinical Case

This material is available as part of the online article from: *link*

This supplementary video is edited using VLLO version 13.6.0 (Vismosoft, Seoul, Republic of Korea)
